# A seven-lncRNA signature for predicting Ewing’s sarcoma

**DOI:** 10.7717/peerj.11599

**Published:** 2021-06-17

**Authors:** Zhihui Chen, Xinyu Wang, Guozhu Wang, Bin Xiao, Zhe Ma, Hongliang Huo, Weiwei Li

**Affiliations:** 1 Department of Orthopedics, Second Affiliated Hospital of Shaanxi University of Traditional Chinese Medicine, Xianyang, Shaanxi, China; 2 Department of Preventive Medicine, School of Public Health, Nanchang University, Nanchang, Jiangxi, China

**Keywords:** Ewing’s sarcoma, LncRNA, Signature, Prognosis, Survival, GEO, ICGC

## Abstract

**Background:**

Long non-coding RNAs (lncRNAs) are a class of non-coding RNAs with unique characteristics. These RNA can regulate cancer cells’ survival, proliferation, invasion, metastasis, and angiogenesis and are potential diagnostic and prognostic markers. We identified a seven-lncRNA signature related to the overall survival (OS) of patients with Ewing’s sarcoma (EWS).

**Methods:**

We used an expression profile from the Gene Expression Omnibus (GEO) database as a training cohort to screen out the OS-associated lncRNAs in EWS and further established a seven-lncRNA signature using univariate Cox regression, the least absolute shrinkage, and selection operator (LASSO) regression analysis. The prognostic lncRNA signature was validated in an external dataset from the International Cancer Genome Consortium (ICGC) as a validation cohort.

**Results:**

We obtained 10 survival-related lncRNAs from the Kaplan-Meier and ROC curve analysis (log-rank test *P* < 0.05; AUC >0.6). Univariate Cox regression and LASSO regression analyses confirmed seven key lncRNAs and we established a lncRNA signature to predict an EWS prognosis. EWS patients in the training cohort were categorized into a low-risk group or a high-risk group based on their median risk score. The high-risk group’s survival time was significantly shorter than the low-risk group’s. This seven-lncRNA signature was further confirmed by the validation cohort. The area under the curve (AUC) for this lncRNA signature was up to 0.905 in the training group and 0.697 in the 3-year validation group. The nomogram’s calibration curves demonstrated that EWS probability in the two cohorts was consistent between the nomogram prediction and actual observation.

**Conclusion:**

We screened a seven-lncRNA signature to predict the EWS patients’ prognosis. Our findings provide a new reference for the current prognostic evaluation of EWS and new direction for the diagnosis and treatment of EWS.

## Introduction

Ewing’s sarcoma (EWS) is a rare but *clinically* significant solid tumor that primarily affects children, adolescents and young adults (AYAs), with an estimated 1.5 cases per million children and AYAs worldwide (Grünewald et al., 2018). About one in four EWS cases occur in soft tissue rather than bone, and about one in four patients with EWS have detectable metastases at the time of diagnosis (Toomey, Schiffman & Lessnick, 2010). Generally, most EWS patients exhibit tumor-related symptoms such as pain or tissue mass (Balamuth & Womer, 2010). The long-term survival rate of patients has not significantly improved (Li et al., 2015) despite efforts to advance EWS treatment strategies, including irritation therapy and surgery. EWS patients’ 5-year survival rate is less than 30% once metastasis has occurred and this rate has not changed significantly over the past 30 years (Amaral et al., 2014). It is vital to identify novel biomarkers to diagnose the disease and predict EWS cases’ prognosis.

Long non-coding RNAs (lncRNAs) are a recently-defined family of transcripts greater than 200 nucleotides in length (Hu et al., 2014). More than 60,000 lncRNAs have been identified in humans and the number is rapidly increasing (Chi et al., 2019). LncRNAs play a crucial role in regulating gene expression though chromatin modification and remodeling, histone modification, and nucleosome localization changes (Deans & Maggert, 2015). Only a few lncRNA functions have been annotated to date. LncRNAs can regulate the survival, proliferation, invasion, metastasis, and angiogenesis of cancer cells (Sahu, Singhal & Chinnaiyan, 2015). Many lncRNAs are reportedly related to cancer progression and patient prognosis. Colon cancer-associated transcript 1 (CCAT1) is highly expressed in colon cancer tissues when compared to adjacent normal tissues. Increased CCAT1 expression has been associated with clinical stage, lymph nodes metastasis, and survival time after surgery (He et al., 2014). HOX transcript antisense RNA (HOTAIR) expression is prognostically significant in patients with gallbladder cancer (Ma et al., 2014), small cell lung cancer (Ono et al., 2014), and breast cancer (Zhang et al., 2016). LncRNAs have gene specific regulatory functions in living cells, and also have a cell type-specific expression pattern corresponding to their mRNA targets, reflecting the cell type’s characteristic biological functions (Chen et al., 2018). Thus, lncRNAs are a type of non-coding RNAs with unique characteristics and tissue specificity, which may have the potential to become diagnostic and prognostic markers. They may also be potential targets for innovative treatment strategies. For example, lncRNA PCA3s can be detected in urine and are specific to prostate cancer. Detecting this RNA has advantages over the widely used serum-based prostate cancer biomarker PSA (prostate-specific antigen) because it is a noninvasive method for finding prostate cancer (Wu et al., 2017). The specificity of lncRNA tissue has been used to selectively kill tumors without affecting normal tissue. Several small-molecule inhibitors created from lncRNAs have been approved by the FDA and have well defined toxicity, body distribution, pharmacokinetics, and pharmacodynamics data (Chandra Gupta & Nandan Tripathi, 2017).

In this study, we analyzed EWS patients’ expression profile data and clinical information using data from GEO. The Kaplan–Meier (KM) estimator and receiver operating characteristic (ROC) curves were applied to screen out differentially expressed lncRNAs related to the diagnosis and survival of EWS patients. Next, the least absolute shrinkage and selection operator (LASSO) algorithm was used to determine key lncRNAs. Finally, we developed an independent prognostic prediction model with seven key lncRNAs and further validated the utility of the lncRNA signature using an external dataset.

## Materials and Methods

### Data acquisition

We obtained an EWS gene expression profile from the Gene Expression Omnibus (GEO) public repository (https://www.ncbi.nlm.nih.gov/gds/) to use as the training cohort and from the International Cancer Genome Consortium (ICGC) portal (https://dcc.icgc.org/releases/current/Projects/BOCA-FR) to use as the validation cohort. The GSE17679 dataset (Savola et al., 2011) contained 106 samples, including 18 normal tissue samples and 88 tumor samples (excluding 11 EWS cell line samples), with clinical information (S1). This dataset was analyzed based on the GPL570 platform (Affymetrix Human Genome U133 Plus 2.0 Array). The ICGC cohort contained 57 EWS tumor samples with their corresponding clinical features (S2). The ICGC’s annotation file and our lncRNA re-annotation file were created using the human reference genome (GRCh38.p12; https://www.ncbi.nlm.nih.gov/genome).

### Differential expression analysis

To obtain differentially expressed lncRNAs (DELncs) from the re-annotated expression profile data, we used the limma R package for differential expression analysis with the following cutoff criteria: fold change >2 and adjusted *P* < 0.05. We applied the “plot” and “pheatmap” packages in R to draw volcano plots and hierarchical clustering plots of the DELncs. The identified DELncs and their expression values were used for subsequent analysis.

### Survival analysis and ROC analysis

Kaplan–Meier curves were drawn using the “survival” R package and a log-rank test was used to analyze the survival rate of all lncRNAs in the training cohort. The ROC curves were drawn using the “survivalROC” R package and the area under the curve (AUC) value was used to analyze the diagnostic value of these lncRNAs (Le, 2019; Le et al., 2021; Le et al., 2019). We set the screening criteria so that the log-rank was *P* < 0.05 and AUC >0.6. AUC values ranged from 0.5 to 1.0, with 0.5 indicating a random probability and 1.0 indicating the perfect ability. It is generally considered that AUC >0.6 as a screening standard has a good predictive value (Li et al., 2020; Song et al., 2020).

### LncRNA signature construction

The hazard ratio (HR) of univariate Cox regression analysis was performed to construct the forest map and further develop the lncRNA signature by using the LASSO regression analysis available in the “glmnet” package. A penalty function in Lasso can be used to build a more accurate model; this method can reduce some nonsignificant indicators to zero by compressing some coefficients to zero and will only retain a small number of indicators with non-zero weight. Finally, the prognostic lncRNAs were selected to construct a risk formula for risk score. The risk score formula was: βlncRNA 1 × lncRNA1 expression + βlncRNA 2 ×lncRNA2 expression +⋯ + βlncRNAn × lncRNAn expression (S3).

### Validating and evaluating the lncRNA signature

We calculated the risk scores of GEO and ICGC patients using the same formula and grouped EWS patients into the high-risk or low-risk group with the corresponding median risk score as the cutoff point. The survival fraction of groups was compared using log-rank test. The sensitivity and specificity of survival prediction according to lncRNA risk scores was determined using ROC curve analysis (S4). A *P*-value <0.05 was considered significant.

### Estimating independent prognostic parameters and nomograms

We used age, sex, and risk score to perform univariate and multivariate analysis with the Cox-regression model on the training and validation groups to assess the prognostic performance of the lncRNA risk scores and to explore the potential prognostic values of these clinicopathological features. Univariate analysis variables were entered into multivariate regression analysis using the stepwise method. Similarly, the time dependent receiver operating characteristic (ROC) curves were used to evaluate the predictive specificity and sensitivity of the risk score. We constructed two nomograms using multivariate regression analysis in the training and validation groups. The predictive accuracy of the nomograms was evaluated using the calibration curves.

## Results

### DELncs identification

**Figure 1:**
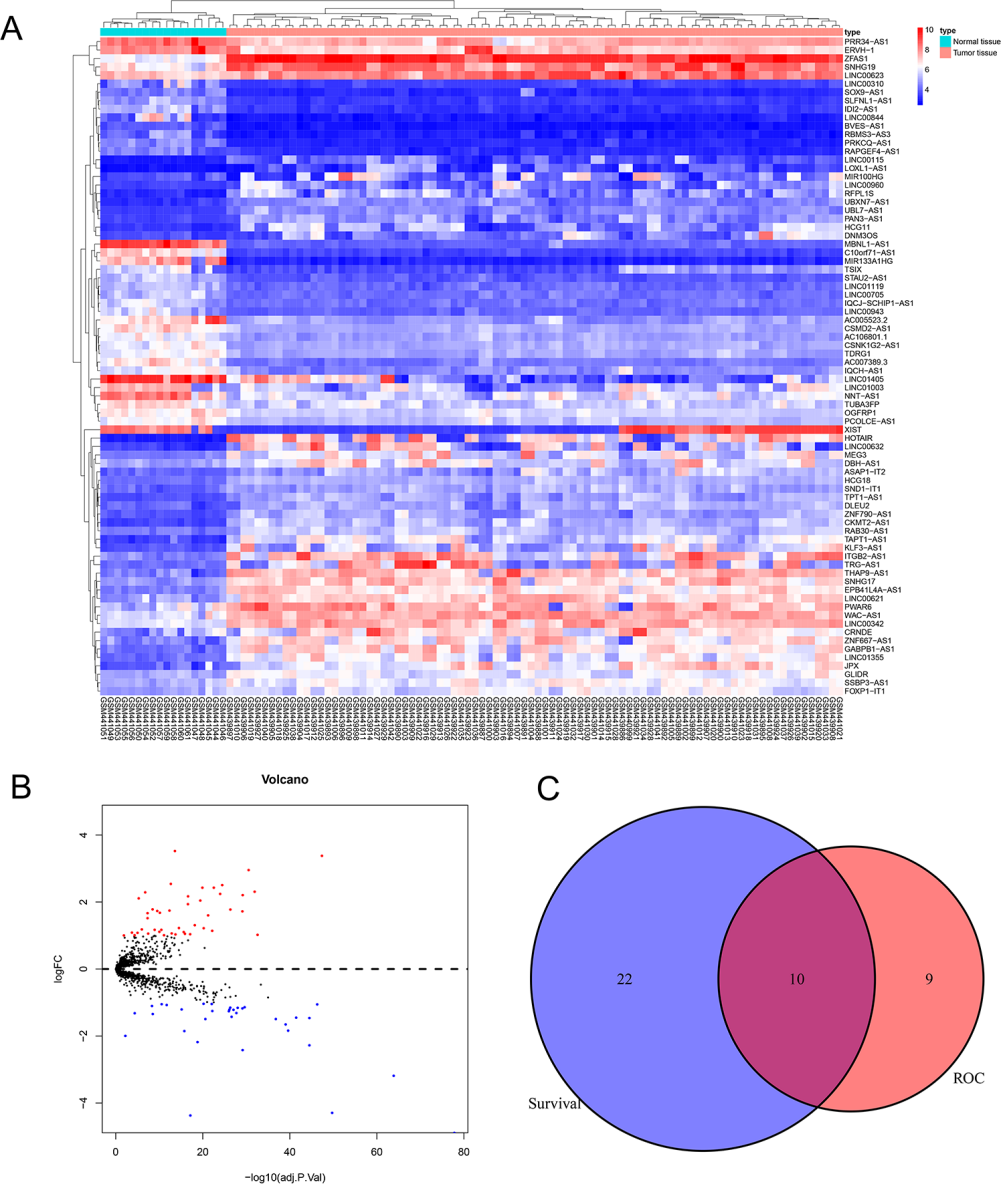
Analysis of differentially expressed lncRNAs related to the EWS. (A) Heat map of the differentially expressed lncRNAs of EWS. (B) A volcano plot of the lncRNAs differentially expressed between the normal tissue and tumor group. The red dots represented the upregulation of lncRNAs (log2FC > 1 and adjusted *P* < 0.05). The blue dots represented the downregulation of lncRNAs (log2FC < − 1 and adjusted *P* < 0.05). C showed that there are 10 common lncRNAs in the intersection of the survival analysis and ROC analysis of EWS patients.

We downloaded expression profile data from the GEO database and obtained 1,146 lncRNAs, which were used to identify candidate EWS-related lncRNAs. We used the “limma” R package to analyze their differential expressions (—log2FC—>1, adjusted *P* < 0.05) and our analysis resulted in 78 DELncs, among which 44 DELncs were upregulated and 34 DELncs were downregulated. We plotted all DELncs in heat maps and volcano maps (Figs. 1A and 1B).

### Diagnostic and prognostic analysis of all lncRNAs in EWS

**Figure 2:**
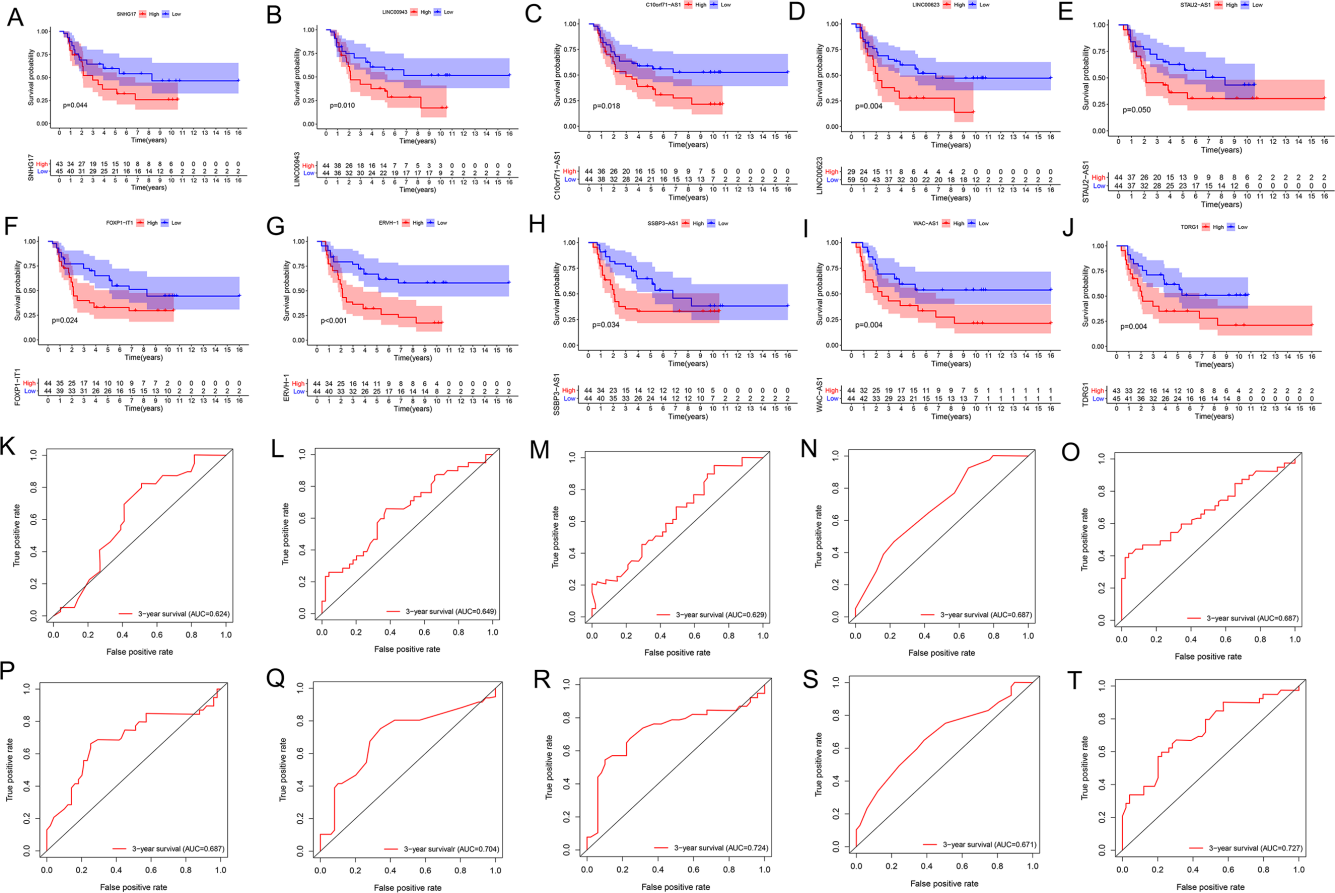
K–M and ROC curves of 10 lncRNAs based on GSE17679 dataset. (A–J) Survival analysis curves of 10 lncRNAs. (A) SNHG17; (B) LINC00943; (C) C10orf71-AS1; (D) LINC00623; (E) STAU2-AS1; (F) FOXP1-IT1; (G) ERVH-1; (H) SSBP3-AS1; (I) WAC-AS1; (J) TDRG1. The red lines represented the high expression of lncRNAs in cancer, and the blue lines represented low expression of lncRNAs. The *X*-axis represented the total survival time (year) and the Y- axis represented the survival rate. (K–T) represented the ROC curves in the order of the above 10 lncRNAs.

We performed the Kaplan Meier survival analysis and ROC curve analysis to evaluate the prognostic and diagnostic value of all DELncs in EWS patients. Ten overlapping lncRNAs were found between DELncs of survival analysis (*P* < 0.05) and ROC analysis (AUC >0.6) (Fig. 1C). Our results showed that 10 key lncRNAs were significantly associated with overall survival (OS) (Figs. 2A–2J) (SNHG17, log-rank test *P* = 0.044; LINC00943, *P* = 0.010; C10orf71-AS1, *P* = 0.018; LINC00623, *P* = 0.004; STAU2-AS1, *P* = 0.050; FOXP1-IT1, *P* = 0.024; ERVH-1, *P* = 0.00022; SSBP3-AS1, *P* = 0.034; WAC-AS1, *P* = 0.004; TDRG1, *P* = 0.004). These key lncRNAs had the following 3-year AUC values: 0.624, 0.649, 0.629, 0.687, 0.687, 0.687, 0.704, 0.724, 0.671 and 0.727, respectively.

### Construction of the lncRNA signature of EWS

**Figure 3:**
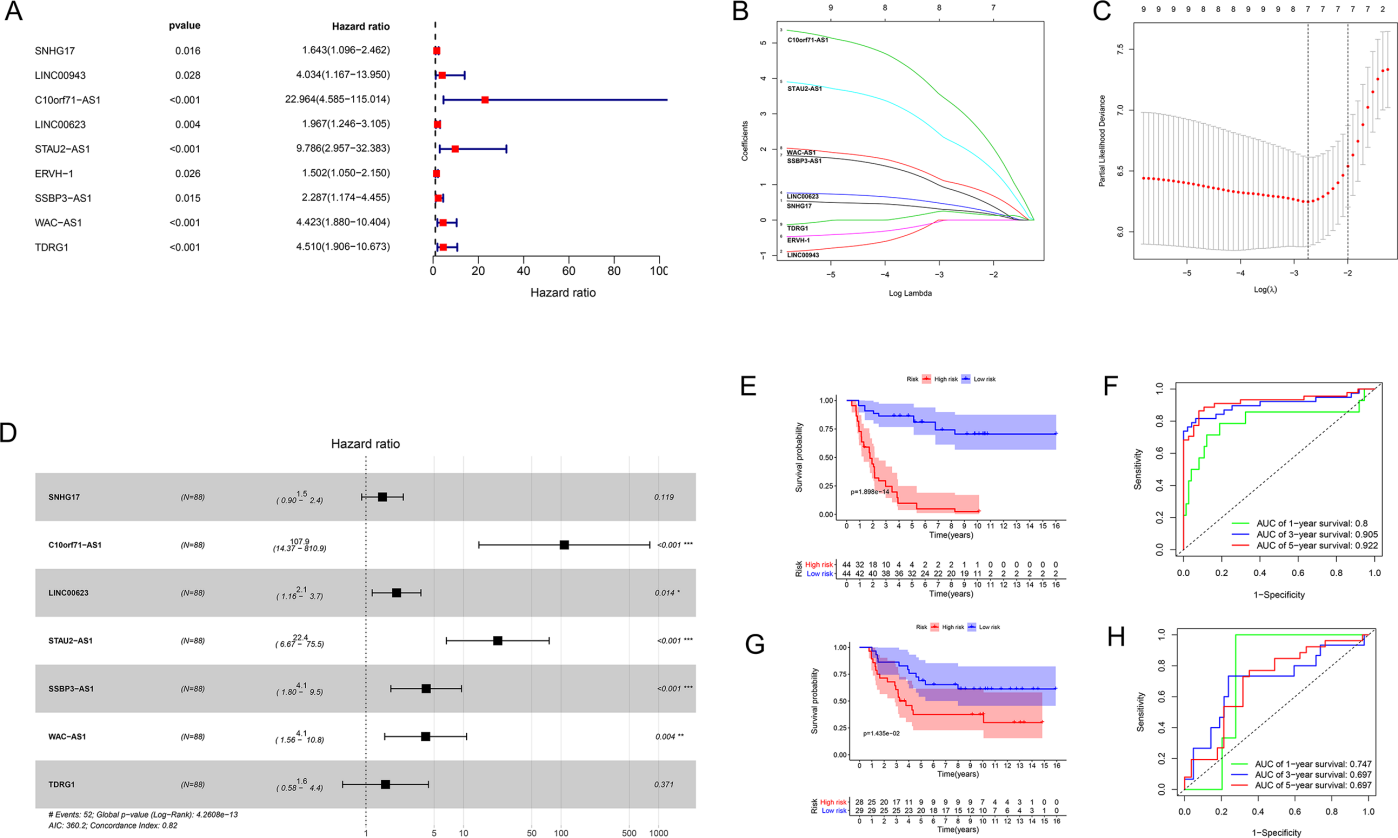
The results of Cox regression and LASSO regression analysis. (A) The forest plot for univariate Cox regression analysis identified nine lncRNAs associated with OS. (B and C) The results from the Lasso regression indicated that all seven lncRNAs were essential for modeling. (D) Forest map of the seven prognostic lncRNAs by univariate Cox regression. (E and G) Kaplan–Meier curves of the training group (E) and validation group (G) showing OS in the low- and high-risk groups classified based on the median risk score. (F and H) ROC curve analyses of the training group (F) and validation group (H) based on the seven-lncRNA signature.

**Figure 4:**
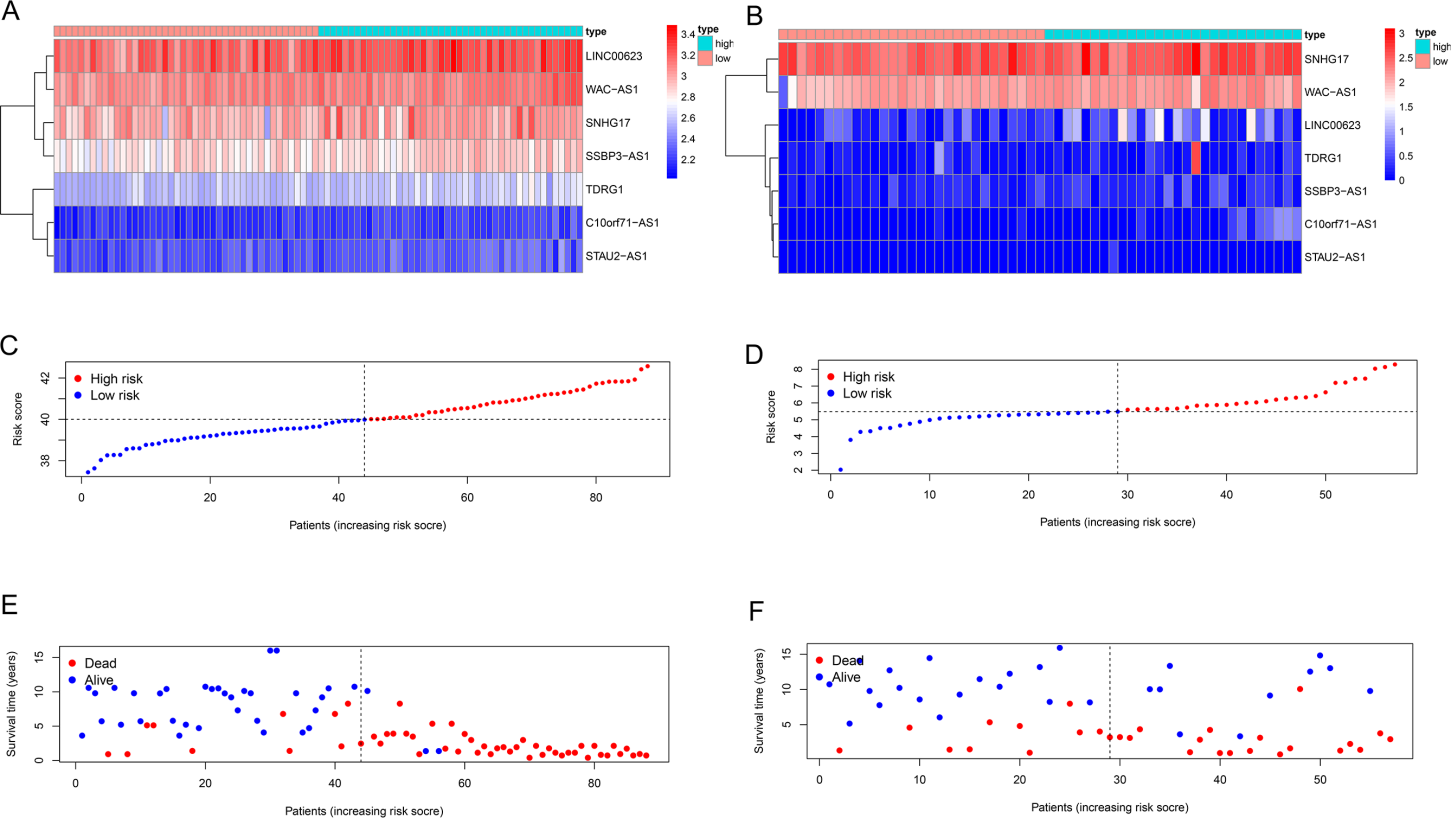
Seven-lncRNA signature risk score in the two groups. (A and B) Heatmap for the seven lncRNAs expression in the training group and validation group. (C and D) Distribution of patients with different risk scores in the two groups. (E and F) Survival status of patients with different risk scores in the two groups.

We used univariate Cox regression analysis of the key lncRNAs to screen nine lncRNAs associated with OS (*P* < 0.05). In the process of constructing the lncRNA prognostic model by LASSO regression analysis, we found that the number of independent coefficients approached zero as lambda increased (Figs. 3B–3D). We performed a ten-fold cross validation procedure for model validation. The confidence interval (CI) under each lambda was analyzed as shown in Fig. 3C. The following prognostic risk score was calculated: (0. 281712273 × SNHG17 expression) + (3. 255312454 × C10orf71-AS1 expression) + (0. 421134052 × LINC00- 623 expression) + (2. 197657636 × STAU2-AS1 expression) + (0. 845771530 × SSBP3-AS1 expression) + (1. 051868412 × WAC-AS1 expression) + (0. 230113692 × TDRG1 expression). EWS patients from the training cohort were categorized into a low-risk group or a high-risk group based on the median value of the risk score. We used the model formula and the seven lncRNAs’ expression to obtain risk scores for each of the 57 EWS samples in the training set. We then calculated the median value of all samples’ risk scores. We compared the median value with the risk score of each sample to obtain a high- and low-risk group. Similarly, we used the model formula and the expression of seven lncRNAs in the validation set to produce a high- and low-risk group. We compared the survival of the low-risk group with that of the high-risk group and found that the survival time of the high-risk group was significantly shorter than the low-risk group (Fig. 3E). We generated ROC curves to assess the prognostic accuracy of the model. The AUC was 0.8 (1-year), 0.905 (3-year), and 0.922 (5-year) (Fig. 3F). The distribution of EWS patients’lncRNA expression, risk score and survival duration were analyzed independently for the training set (Figs. 4A, 4C, and 4E).

### Validating the seven-lncRNA signature for prognostic evaluation

We used the same coefficients in the validation dataset to determine the robustness of this model. We classified patients with a high-risk (*n* = 28) and a low-risk (*n* = 29) into groups taking the median score as the cutoff point; we used the same risk formula in the ICGC cohort (n = 57). The KM survival curve showed that the OS rate was significantly worse in the high-risk group compared with the low-risk group (*P* value = 0.01425; Fig. 3G). Heatmaps depicting risk lncRNA expression, risk score distribution plot, and survival status plot of the different risk groups in the ICGC cohort are shown in Fig. 4B, D, and F. The AUC values in this validation cohort were 0.747 (1-year), 0.697 (3-year), and 0.697 (5-year) (Fig. 3H).

### Evaluation of the risk model as independent prognostic factor for EWS

**Figure 5:**
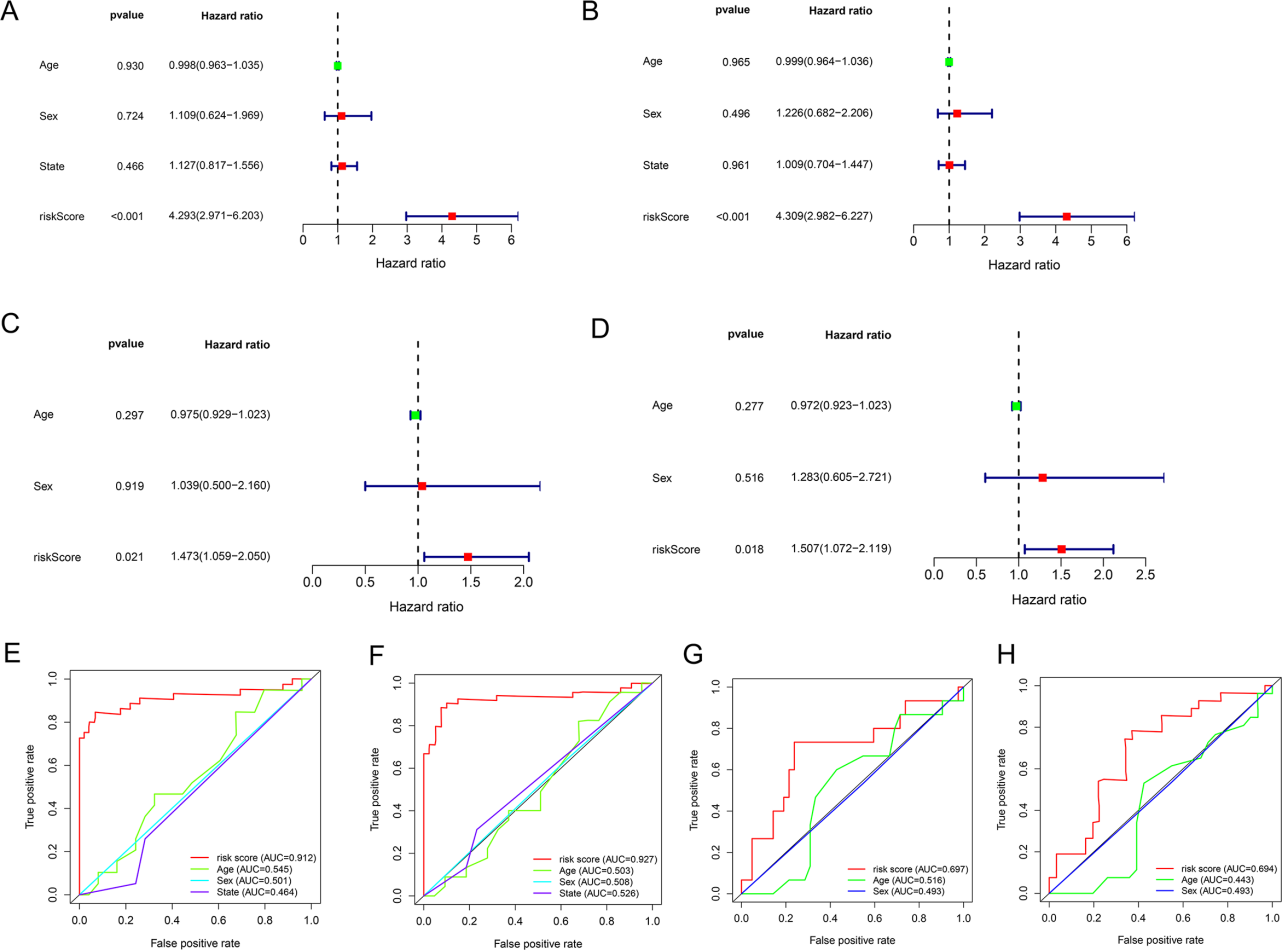
The results of univariate and multivariate Cox regression analyses for clinicopathological factors influencing OS. (A and C) Univariate Cox regression analyses to estimate the clinical factors that influence OS in the training (A) and validation (C) group. (B and D) Multivariate Cox regression analyses to estimate the clinical factors that influence OS in the training (B) and validation (D) group. (E and G) The 3-year AUC for risk model score and clinical features according to the ROC curves in the training (E) and validation (G) group. (F and H) The 5-year AUC for risk model score and clinical features according to the ROC curves in the training (F) and validation (H) group. Clinical features: Age and Sex.

**Figure 6:**
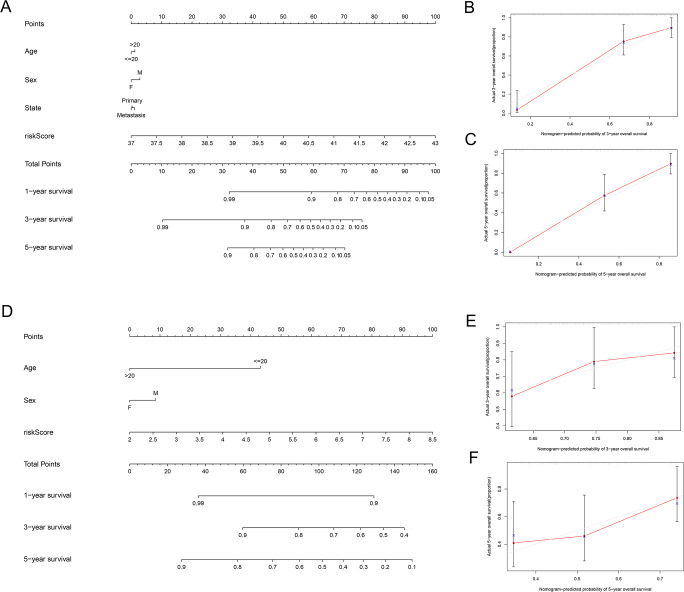
Establishment of two nomograms for OS prediction in EWS. (A and D) Nomograms integrating the risk score, age, and sex based on the seven-lncRNA signature in the training (A) and validation (D) group. (B, C, E and F) Calibration curves of the two nomograms. (B and E) Calibration curves for predicting patients survival at 3-year in the training (B) and validation (E) group. (C and F) Calibration curves for predicting patients survival at 5-year in the training (C) and validation (F) group.

Univariate and multivariate Cox regression analyses were performed to assess whether the lncRNA signature-based risk score was an independent prognostic factor for EWS. The HR of the risk score and 95% CI were 4.293, 1.473, and 2.971–6.203, 1.059–2.050 (*p* < 0.001, *p* = 0.021) in the univariate Cox regression analysis of the training cohort (Fig. 5A and 5C). The HR of the risk score and 95% CI in multivariate Cox regression analysis were 4.309, 1.507 and 2.982–6.227, 1.072–2.119 (*p* < 0.001, *p* = 0.018 (Fig. 5B and 5D) for the validation cohort. The risk model of the seven lncRNAs was the most significant prognostic factor for EWS, independent of clinicopathological parameters such as age and sex. We estimated the area under the ROC curve of the risk score to evaluate the predictive specificity and sensitivity of the risk score on the prognosis of EWS patients. The 3-year AUC values of the risk score for the training and validation cohorts were 0.912 and 0.697, respectively (Fig. 5E and 5G). The 5-year AUC values of the risk score were 0.927 and 0.694, respectively (Fig. 5F and 5H), followed by the AUC of age and sex. These results indicate that the prognostic risk model for the seven lncRNAs for EWS was reliable. The results indicate that the seven-lncRNA signature was a significant independent prognostic factor for EWS patients. Two nomograms of the training group (Fig. 6A) and validation group (Fig. 6D) were constructed based on the prognostic signature and clinical factors, such as age and sex. The calibration plots showed good agreement in predicting OS with the actual probability of OS at 3- and 5-year in the training (Fig. 6B and 6C) and validation cohorts (Fig. 6E and 6F).

## Discussion

EWS is one of the most common malignancies in children. Approximately 70% of EWS children can be cured by surgery and chemotherapy, regardless of whether they receive radiation therapy, however, only 30% of metastatic cancers can be cured (Sun et al., 2017). LncRNAs were initially considered to be transcription noise. The role of lncRNA in human diseases has been recognized recently, especially in human cancer (Peng, Koirala & Mo, 2017). LncRNA is involved in tumor differentiation, proliferation, metastasis, and transcriptional regulation. A large number of studies have reported that lncRNA signatures were related to the development and prognosis of cancer (Li et al., 2019; Dinescu et al., 2019). However, the research on lncRNAs in EWS is limited and we sought to screen the lncRNA signature to aid in the diagnosis and treatment of this disease.

We used the training cohort to screen survival-related DELncs in the EWS, and 10 lncRNAs were significantly-associated with the survival of patients with EWS (log-rank test *P* < 0.05; AUC >0.6). Univariate COX regression and LASSO analyses were used to identify seven prognosis-related lncRNAs (SNHG17, C10orf71-AS1, LINC- 00623, STAU2-AS1, SSBP3-AS1, WAC-AS1, TDRG1) (*P* < 0.05). The seven-lncRNA signature had a high predictive value of overall survival in the training and validation cohorts. The 1-, 3-, 5-year AUC values of ROC showed that this model had superior accuracy. Two nomograms, including the seven-lncRNA signature, age, and sex were established to predict EWS prognosis in the two cohorts. The ROC analysis results showed that the prognostic risk model of the seven lncRNAs for EWS was reliable. We found that the high expression group had a low probability of survival. Increased expression of SNHG17, WAC-AS1, LINC00623, SSBP3-AS1, and TDRG1 caused the risk score of patients to increase, indicating a poor prognosis. Of these seven lncRNAs, none has been related to EWS disease and additional studies are needed to verify them in the future. The small nucleolar RNA host gene 17 (SNHG17) has a length of 1,186 bp and belongs to a large family of noncoding genes hosting small RNAs (Zhang et al., 2019; Han et al., 2020). Previous studies have proven that SNHG17 is highly-expressed and carcinogenic in cancers, including melanoma, gastric cancer, and colorectal cancer (Wu et al., 2020; Gao, Liu & Sun, 2019). The lncRNA testis developmental related gene 1 (TDRG1) has been identified as a proto-oncogene for many tumor types, including gastric carcinoma, cervical cancer, epithelial ovarian cancer, endometrial cancer, and testicular germ cell tumors (Ma et al., 2020; Peng et al., 2019). LINC00623 also plays a role in hormone-related cancers (Wang et al., 2020).

Our study was limited by its reliance on a GEO dataset with a small sample size to establish the lncRNA prediction model. The small sample size may indicate that the expression levels between the high- and low-risk groups are similar. The expression levels of some lncRNAs (such as LINC00623, SSBP3-AS1, and TDRG1) are different between the training group and the validation group. The number of samples may not produce consistent results for bioinformatics analysis and additional samples are needed to validate the prognostic performance of our proposed lncRNA signature for EWS. In addition, a luciferase reporter assay and chromatin immunoprecipitation should be applied to the lncRNAs to verify their expression levels, biological functions, and specific regulatory mechanisms in EWS.

We constructed and validated a seven-lncRNA signature to predict the prognosis of patients with EWS. Our findings provide a new reference for the prognostic assessment of EWS and may help in the diagnosis and treatment of EWS in the future.

##  Supplemental Information

10.7717/peerj.11599/supp-1 Supplemental Information 1 Risk score file of the GEO 88 EWS patients with clinical information and risk score in the training cohort.

10.7717/peerj.11599/supp-2 Supplemental Information 2 Risk score file of the ICGC 57 EWS patients with survival information and risk score in the validation cohort.

10.7717/peerj.11599/supp-3 Supplemental Information 3 Code to build and verify the model The script was written in R using the glmnet and survival packages.

10.7717/peerj.11599/supp-4 Supplemental Information 4 Code for ROC analysis The script was written in R using the survival, survminer and timeROC packages.

## Additional Information and Declarations

### Microarray Data Deposition

The following information was supplied regarding the deposition of microarray data: The datasets are available at Gene Expression Omnibus (GSE17679) and the International Cancer Genome Consortium (ICGC) database (https://dcc.icgc.org/relea- ses/current/Projects /BOCA-FR).

### Data Availability

The following information was supplied regarding data availability: The original data are available in the Supplemental Files.
